# The Atlanta Meeting of the American Medical Association

**Published:** 1896-06

**Authors:** 


### Editorial.

### THE ATLANTA MEETING OF THE AMERICAN MEDICAL ASSOCIATION.

As a resident of Atlanta, the editor has prepossessions in its favor that render it more becoming to give what our exchanges have to say of the success of the meeting, than to describe it ourselves:

*The American Review:*—The annual meeting of the American Medical Association has been a most successful one.

*The Philadelphia Polyclinic:*—The Atlanta meeting of the American Medical Association passed most enjoyably and busily. So crowded with good section work are all of the meetings now that those attending one section faithfully had little opportunity to follow the general course of events. The weather was most propitious, the registration met all reasonable expectations, and the programmes of the sections were full and interesting enough to tempt men from their due allegiance and make them wanderers through all the specialities. The hospitalities extended were most kind and typical; the open clubs, the Georgia barbecue and the beautiful receptions at the homes of the eminent practitioners along the famous Peachtree street, most acceptable in themselves and having the great, though negative, merit of interfering very little with the scientific work.

*The Journal of the American Medical Association:*—There have been few meetings in the history of the Association which have passed off more harmoniously and more pleasantly in all respects than that just concluded at Atlanta. . . . The places of meeting provided for the sections at Atlanta were ample and easy of access. Socially the Southern hospitality, which is always conspicuous at these gatherings, made itself felt in such a way as to make every delegate feel at home. The visit to the charming home of the Thompsons at Brookwood, the Georgia barbecue, and the receptions at the homes of Drs. Calhoun, Ridley and Todd, are occasions long to be remembered. Indeed if there is any criticism it is that Dr. Westmoreland and the committee of arrangements and citizens did too much to make the visit of the members agreeable and pleasant. Truly the forty-seventh annual meeting of the association should be marked with a white stone as one of its *Dies Memorabiles*.

*The Cincinnati Lancet-Clynic:*—The hospitalities of the occasion were excellent. A unique feature was the barbecue given Wednesday afternoon which was much enjoyed. Private receptions in the evening of Thursday, opened some of the mansions of the city; they are superb, and their occupants are masters in the art of hospitality. The city in all of its clubs, public and private buildings was wide open to all delegates.

Atlanta impresses the Northern visitor very favorably, and is destined to become a great commercial metropolis. Its business relations with Cincinnati are exceedingly intimate, and must ever remain so. . . . Altogether, we had a good time, enjoyed the outing, most of the meetings and greetings of scores of old and devoted friends.

These extracts are sufficient to show that as far as Atlanta and the work of the local committee are concerned, the meeting was an unqualified success. It is a source of gratification to the local profession to know this, as the task that they had voluntarily assumed was a large one, and was begun not without misgivings.

Among the many valuable papers read at the meeting none were more interesting than the addresses of Dr. Wm. Osler, on "The Study of Fevers in the South," Dr. Nicholas Senn, on "Some of the Limits of the Art of Surgery," and that of Dr. Geo. H. Rohe, on "The Subject of the Purification of Water Supplies."

Dr. Osler said that humanity has three great enemies, fever, famine and war. We have added greatly to our knowledge of fever, but the improvement in treatment has not kept pace. Typhoid fever is essentially a disease of the country. Dr. Osler had no patience with the antiseptic and so-called abortive treatment of typhoid fever. We should gain more knowledge by holding autopsies whenever possible. We young physicians of the South should study the cause of malaria fever, the standpoint of Lavaran's discovery of the plasmodium.

Surgery was approaching the dignity of a science. Pathology and bacteriology are the foundation of the advances of surgery. Antiseptic and aseptic surgery had made easier the path of the practical surgeon, and have nearly eradicated those of the older surgeon's greatest enemies, hospital gangrene, erysipelas and secondary hemorrhage, and have minimized the occurrence of suppuration and its complications. Operative surgery has been carried to extremes.

Phlegmonous processes had been lessened in frequency by the antiseptic methods. Injections of carbolic acid and the internal use of alcohol in heroic doses, promised most in their treatment.

Early operative interference was indorsed in osteomyelitis. In tuberculosis of the joints, intra-articular and parenchymatous, injections of iodoform glycerine have given more satisfactory results than resection or orthorectomy. On the subject of malignant tumors, he summed up the modern treatment as follows: "Operate early and thoroughly."

Dr. Senn gave it as his opinion that operation was indicated in most cases of fracture of the skull.

Surgery of the stomach for malignant disease was not encouraging. Dr. Senn made a plea for conservatism in gynecological surgery, particularly in the matter of castration of women.

The Ramm-White operation of removal of the testicles for non-malignant obstructive enlargement of the prostrate deserved a fair trial, but the author has been apprehensive of evil in the future, not so much from the proper use as from the abuse of the procedure.

Dr. George H. Rohe, in his address on State Medicine, entitled, "The Purification of Public Water Supplies," said that to obtain purity we must endeavor to prevent contamination of water with impurities, or else purify the water after it had become polluted. The latter is the more practicable, and can be accomplished by filtration, preferably by sand. The rest of his paper was devoted to a consideration of the effectiveness and proper methods of construction of filters.

Besides the meeting of the American Medical Association, the American Academy of Medicine held its annual gathering May 2d and 4th, at which many interesting papers were read. The following bodies also met at the same time: Medical Publishers' Association, National Confederation of State Medical Examiners and Licensing Board, Georgia Pharmaceutical Association, Southern Railroad and Alabama Great Southern Railroad Surgeons, American Editors Association.

